# Acoustic Detection of Insects in Stored Products in the Presence of Strong Ambient Noise

**DOI:** 10.3390/s26051511

**Published:** 2026-02-27

**Authors:** Daniel Kadyrov, Alexander Sutin, Nikolay Sedunov, Alexander Sedunov, Hady Salloum

**Affiliations:** 1 Department of Civil, Environmental & Ocean Engineering, Stevens Institute of Technology, Hoboken, NJ 07030, USA; asutin@stevens.edu (A.S.);; 2 Department of Civil Engineering, University of Houston, Houston, TX 77204, USA

**Keywords:** acoustic, stored product, insect, rice

## Abstract

Acoustic detection methods offer a non-destructive alternative to manual inspection for identifying insect infestations in stored products, but their performance is compromised by ambient noise in operational environments. This study presents an enhanced detection algorithm for the Acoustic Stored Product Insect Detection System (A-SPIDS) that enables reliable single-insect detection in the presence of strong external noise. The platform’s physical noise isolation achieved an average attenuation of 45 dB above 2000 Hz. Spectral analysis revealed that insect signals dominate over ambient noise, generating insect-like impulses in the high-frequency band, enabling optimization of the Normalized Signal Pulse Amplitude (NSPA) detection metric to the 1565 Hz to 6000 Hz frequency band, resulting in 99.4% detection accuracy at 80 dBA ambient noise levels. The external microphone was leveraged to identify and remove noise-generated impulses from internal piezoelectric sensor recordings, achieving 100% detection with zero false alarms across the recorded dataset featuring species *Callosobruchus maculatus*, *Tribolium confusum*, and *Tenebrio molitor* in oatmeal, rice, wheat, and corn products at noise levels exceeding 100 dBA.

## 1. Introduction

### 1.1. Stored Products and Stored Product Insects

Stored products refer to dried, durable raw agricultural and processed commodities such as grains, cereals, seeds, nuts, and dried fruits that are kept for extended periods and make up the largest percentage of plant-based food [1]. These products are susceptible to infestation by stored product insects during farming, storage, and transportation.

Insects attack stored products in a variety of ways and are categorized by their eating habits as internal feeders, external feeders, and secondary pests. The rice weevil, *Sitophilus oryzae*, the granary weevil, *Sitophilus granarius*, and the lesser grain borer, *Rhyzopertha dominica*, are an example of internal feeders, which bore into the kernels of grains and seeds as larvae and feed on the endosperm, visually undetectable, until they emerge as adults, leaving burrowing holes and powder residue. External feeders, such as the drugstore beetle, *Stegobium paniceum*, the cigarette beetle, *Lasioderma serricorne*, and the Khapra beetle, *Trogoderma granarium*, chew through the outer seed coat of the grain exterior and then devour the inside. The red flour beetle, *Tribolium castaneum*, and the sawtoothed grain beetle, *Oryzaephilus surinamensis*, are examples of secondary pests, which feed on previously damaged, processed, or broken-down material such as flour, meal, and bran. The mealworm, *Tenebrio molitor*, is also considered a secondary pest and additionally feeds on damp, molded, or decomposing material [2,3].

An unmitigated infestation can lead to significant ecological, agricultural, and economic impact. Post-harvest insects are responsible for 10% to 20% of storage losses [4]. Beyond direct loss, the insects can introduce pathogens and allergens into the material, reduce the quality of the product by changing its chemical and nutritional composition, and produce metabolic artifacts that further degrade it. Invasive species, such as the Khapra beetle, can enter new regions and disrupt the local ecosystem and economy. The presence of insects in the material can also lead to the rejection of the product by the consumer, the importer, or the regulatory agency [1,2,4,5].

### 1.2. Methods for Stored Product Insect Detection

According to the International Standards Organization (ISO) standard on the detection of insects in cereals and milled products, a container is deemed infested if a single insect is found within 1 kg of material [6]. The main method employed at United States Ports of Entry to inspect stored product for insects is through manual inspection, where the chosen commodity is emptied on an inspection table and personnel, often with tools including sieves, tweezers, magnifying glasses, and intense lights, sift through the material looking for live and dead insects at different life stages, fragments, or signs of insect infestation such as webbing, casings, frass, or burrowing holes in the kernels. This process is time and physically consuming, requiring experienced personnel or with a background in entomology to identify the species of the insect to determine the appropriate course of action. If the insect is identified as a quarantine pest, the material is either treated, destroyed, or rejected and an Emergency Alert Notification (EAN) is issued to the importer [7]. In a recent case, evidence of a Khapra beetle infestation was found by United States Customs and Border Protection (CBP) inspectors in a small bag of undeclared seeds in a carry-on from Lebanon while performing a secondary visual inspection [8].

Various tools and devices have been developed, often leveraging sensing technologies, in order to expedite areas within the inspection, including automating the examination process, detecting insect infestation, or identifying the insect species. There are a multitude of books and reviews that summarize and compare the different methods available [5,9,10,11,12,13,14,15,16].

Physical methods are the most conventional and widely used techniques for detecting insects in stored products. Traps can be placed aerially, on the surface of the material, or inserted into the bulk of the product to capture insects, and are effective for long-term storage [9,17]. When placing traps is difficult, probes can be inserted into the product to measure or collect samples at different depths. Often, visual lures, lights, pheromones, and food attractants are used to increase the effectiveness of the traps [18]. The Berlese-Tullgren trap uses heat and light to drive insects out of a material sample and into a collecting vessel [19]. Although these methods require long durations to collect sufficient samples and cannot detect insects burrowed inside kernels, automated tools have been developed, such as the TNAU Insect Detection System, Insector, and the Electronic Grain Probe Insect Counter (EGPIC), to expedite the counting and identification of the captured insects [12,20,21,22]. Other physical methods include rolling machines that automate the sieving of products, systems that monitor for changes in electrical conductance between whole, damaged, and infested kernels, and the flotation method, which separates possible insects from product by density, but these are relatively more expensive, require material-specific calibration, and tend to damage the material in the process [23,24,25].

Chemical sensors monitor changes in the material composition caused by the presence of insects. An increase in carbon dioxide (${\mathrm{CO}}_{2}$) levels can indicate the presence of insects, fungi, and mites. Although an increased ${\mathrm{CO}}_{2}$ concentration above 500 parts per million (ppm) can indicate insect respiration or pest activity, it cannot specify the type of pest or level of infestation and instead could be used as an early indicator of spoilage [26]. Insects release specific volatile organic compounds (VOCs) such as uric acid, the main element of their excreta, that can be monitored through gas chromatography-mass spectrometry (GC-MS) and Electric-nose (E-nose) systems [20,27,28]. These chemical sensors were able to use the VOCs to identify *Cryptolestes ferrugineus* [29], *Tribolium castaneum* [30], *Rhyzopertha dominica* and *Sitophilus granarius* in wheat samples [31]. The VOC types and levels vary at extreme hot and cold temperatures, so these systems can also validate if treatments were effective [26].

Imaging methods leverage different parts of the light spectrum to visualize and identify insects in stored products. Thermal cameras capture the infrared radiation, or heat signature, emitted by the insects in the material. In some systems, a heating unit or refrigerator is used to alter the temperature of the material to increase the thermal difference between the insects and the stored product [32,33]. The thermogram, a visual representation of the temperature distribution, can be used to detect damaged grains and foreign materials, as well as internal and external infestations [12]. Thermal imaging was also able to detect *Cryptolestes ferrugineus* inside wheat kernels [32]. Near-infrared (NIR) spectroscopy and hyperspectroscopy use the absorption and reflectance of different substances at wavelengths in the range of 780 nm to 2500 nm. This method was able to differentiate between uninfested and infested wheat kernels with *Sitophilus oryzae*, *Rhyzopertha dominica*, and *Sitotroga cerealella* as well as detect internal feeders inside the kernels [34]. X-ray imaging systems, such as the TrapsSesotec RAYCON X-Ray system and the X-ray Inspection System AD-4991, visualize invisible insects within stored food grains as well as fruits and vegetables. They were used to perform real-time inspection of *Sitophilus granarius* in wheat kernels [35]. These spectral methods are rapid and non-destructive, but require expensive equipment and specialized trained personnel to operate and interpret the results. Machine learning models and image recognition algorithms have effectively been used to classify the insect species [12].

Microwave detection methods detect differences between emitted and reflected radar frequencies to identify the movement of insects within stored products. A system, originally designed for termite detection, was used to track the movement of one to twenty adult *Lasioderma serricorne*, *Oryzaephilus surinamensis*, *Attagenus unicolor*, and *Tribolium castaneum* at distances up to 30 cm in corn meal and flour mix. It was also able to quantify the level of infestation [36]. A similar system successfully detected *Tribolium castaneum* and *C. ferrugineus* in wheat grains at distances up to 2.1 cm and 1.2 cm, respectively, while operating within the 3 GHz to 4 GHz frequency range. This method can only detect the movement of the insects and is highly susceptible to vibrational interference [37].

### 1.3. Acoustic Detection of Insects in Stored Product

Acoustic sensors and algorithms leverage the sounds and vibrations generated by insects as they move, communicate, masticate, and procreate within a material. Although many studies refer to these techniques as vibro-acoustic methods, this paper adopts the term “acoustic” to denote elastic waves within the tested materials. Platforms utilizing the sensors can be designed as standalone receptacles where product is placed momentarily during examination, as probes that could be inserted into the product, or mountings for storage containers for continuous monitoring. Beyond the detection of an insect presence, some acoustic systems can also classify the species and life stage, determine the level of infestation, and identify the behavior of the subject. Although acoustic methods are relatively low-cost and non-destructive, they are highly susceptible to external environmental noise and vibration that can limit the detection capabilities by masking the signals generated by the insects and increasing the false positive and negative rates.

Several extensive reviews have been published that summarize the acoustic detection methods for stored product insects [14,16,38,39]. The following subsections review the sensors and hardware design considerations for acoustic detection of stored product insects, the datasets available for training and evaluating machine learning models, the observed vibro-acoustic properties of the subjects in the material, and the available systems capable of automated insect detection.

#### 1.3.1. Sensors for Detection of Acoustic Signals Generated by Stored Product Insects

Traditional microphones convert sound waves in the air into electrical signals. In stored product insect detection, these microphones are often used to capture the sounds emitted by the insects as they displace material, feed, or vocalize. Microphones can be used in a contact manner, such as in the Portable Postharvest Detection System (PDS), which integrated an electret condenser microphone into a plexiglass base on which the grain sample is placed [40] or directly inserted into the material [41]. Other systems separate the microphone from the material through an air gap [42,43,44]. Another application is through impact acoustics which, instead of measuring insect sounds, detects the differences in sounds generated by whole and damaged kernels. Microphones are placed near a metallic plate and differentiate the sounds radiated by the strikes of whole and damaged kernels as they are individually dropped through a single-file feeder [45,46].

Piezoelectric sensors and accelerometers measure the elastic waves generated by insects and propagate through granular materials. Piezoelectric materials convert mechanical stress produced by the elastic waves into electrical signals. The sensors can be integrated into acoustic probes, such as the Early Warning Detector (EWD) [47], which can be inserted into the material [48,49]. Systems such as Acoustic Location Fingerprinting Insect Detector (ALFID) [50] and the Acoustic Stored Product Insect Detection System (A-SPIDS) [51], are designed as standalone receptacles with embedded piezoelectric sensors where the product is placed momentarily during examination. Accelerometers sense the vibrations in up to three orthogonal axes. The TreeVibes system was developed as a probe with a uniaxial accelerometer to detect wood-boring insects in trees [52,53] but was explored to detect *Sitophilus oryzae* eggs, larvae, and adults in 300 mL wheat grains over a weeklong period. Another study considered an accelerometer attached to wheat stems in order to detect *Cephus cinctus* and *Metamasius callizona*, demonstrating its potential usage in wheat pre-processing stages [14,54].

Traditional microphones, piezoelectric sensors, and accelerometers can be extended to support ultrasonic frequencies above 20,000 Hz. A condenser microphone was used to capture the stridulation of *Oryzaephilus mercator*, *Cryptolestes pusillus*, *Sitophilus granarius*, *Anthrenus flavipes*, *Trogoderma glabrum*, *Ephestia elutella*, and *Cadra cautella* in the 15,000 Hz to 60,000 Hz range [55].

The Ultrasonic Insect Detector was patented in 1989. It is an active insect detection system that radiates and receives ultrasonic pulses. It uses a half-inch diameter piezoelectric transducer with a center frequency of 40,000 Hz. The system was used to detect *Callosobruchus maculatus* larvae internally feeding inside cowpeas [56,57].

Although not specifically for stored product insects, ultrasonic emitters and receivers have been studied to detect pests and insect damage in wood by measuring changes in the time of flight (TOF) as well as ultrasonic spectrum attenuation [39].

Advances and increased accessibility to micro-electromechanical systems (MEMS), which miniaturize traditional, piezoelectric, and accelerometer sensors, have enabled the development of compact, low-power, and cost-effective acoustic detection systems [58,59]. These sensors, as well as other elastic wave sensors such as laser Doppler vibrometers (LDV), which measure surface vibrations, have been explored for detecting insects in other materials such as trees, plants, and soil [14,16,38,39,60,61].

#### 1.3.2. Anechoic Chamber Application in Acoustic Insect Measurements

One significant drawback of using acoustic sensing is its vulnerability to external noise, prevalent in expected operation environments such as storage and inspection facilities, which can mask the insect sounds and lead to false positives and negatives [38].

An initial step in limiting the effects of external noise is through the platform design by incorporating shielding, soundproofing, and vibration isolation. Mankin and colleagues [62,63] developed two large and heavyweight acoustic anechoic chambers that were applied for insect signature measurement. The first system reduced background noise by 50 dB to 70 dB from 1000 Hz to 10,000 Hz by shielding a sensor with six layers of alternating plywood, loaded vinyl, and foam wedges totaling 26 cm and resulting in a muffle box weighing 81 kg [62]. The subsequent system incorporated a cylindrical vessel weighing 70 kg and suppressed 60 dB to 90 dB between 1000 Hz and 10,000 Hz using a 1 mm steel wall and 50.8 mm of foam [63].

The other double chamber designs [59] further isolate the sensor from external noise by enclosing the sensor in an inner chamber that is separated from an outer chamber by an air gap. The system described in [41] used a double-wall chamber where a 50 mm air gap separated an inner container lined with three different compressed density foams between two 18 mm plywood walls vertically spring-mounted from the outer container featuring a wall of 50 mm of acoustic foam and 18 mm of plywood.

The system in [44,64] used a spring-suspended double wall chamber to investigate low frequency band and external vibration isolation. It used wood paneling and acoustic foam layers that weighed 620 kg and was capable of sound attenuation of 50 dB above 100 Hz. The microphone array inside this chamber was utilized to develop the InsectSound 100 dataset [44,64]. Additional microphones placed outside the recording platforms have also been leveraged to identify external noise levels that could impact the detection performance [44,63,64].

#### 1.3.3. Acoustic Insect Datasets

Several audio databases featuring insects have been published and are publicly available. The Animal Sound Archive [65], InsectSet32 [66], InsectSet47 and InsectSet66 [67,68], InsectSound1000 [44], and Singing Insects of North America (SINA) [69] all feature insect recordings; however, these datasets do not include species commonly found infesting stored products nor audio recordings of insects within the targeted commodities. BugBytes features 95 recordings of insects in different environments totaling approximately 23 min, with less than 15% pertaining to stored product insects [70].

#### 1.3.4. Acoustic Signatures of Insects in Stored Products

Insect activity manifests in acoustic recordings as impulses, high-magnitude signals across a wide frequency range and short duration. Insects generate sounds within the material during locomotion through friction between the body and the material as well as through the movement of the wings, legs, and other body parts. Sounds are also generated by mastication, communication, and procreation. The production of these sounds is strongly affected by the surrounding material, the insect size and life stage, the density of the infestation, and behavior of the subject, which can vary with temperature and external disturbances [49,71]. The temporal and spectral characteristics of the acoustic signals generated by insects in stored products can be used to identify the presence of insects, classify the species and life stage, and determine the level of infestation.

The temporal domain refers to the time-varying nature of the signal, including amplitude and duration. Examples of pulses generated by the *Callosobruchus chinensis* and *Callosobruchus maculatus* in chickpea and green gram are presented in [43]. A maximal average amplitude of 98 dB was observed for the *Callosobruchus chinensis* in green gram. The pulse durations were in the range of 50 ms to 70 ms. The authors noted that a threshold based on amplitude could be used to detect infestation and discern between the two species [43]. Amplitude variance was also used to quantify the infestation density of *Callosobruchus maculatus* from zero to 500 insects in green gram and cowpeas [59]. Although a single impulse might be in the range of 3 ms to 30 ms, insect bursts are characterized as a series of patterns with a duration of up to 250 ms [14]. By designating a burst as a train of five or more 3 ms to 10 ms impulses separated by 1 s intervals, statistical differences were found between the *Stegobium panicium*, *Tribolium cataneum*, and the *Sitophilus oryzae* [72].

The spectral domain refers to the frequency content of the insect sound. Generally, insect activity occurs in the frequency band in the range of 80 Hz to 8000 Hz and can vary between the species and life stages [38]. A system using two piezoelectric HT378C20 sensors measured male *Trogoderma granarium* producing sounds with dominant frequencies in the range of 3500 Hz to 4500 Hz and from 1000 Hz to 17,000 Hz in their adult and larval stages, respectively, when placed in 200 g of wheat [73]. Female adults of the same species dominated in bands in the range of 1000 Hz to 3000 Hz and 4000 Hz to 5000 Hz. The confused flour beetle, *Tribolium confusum*, larva produced peak frequencies at 2400 Hz when eating flour, while adult insects feeding generated signals at 2000 Hz. Their movement through the material emitted noise at 2300 Hz [74]. Spectral-based algorithms can match the frequency content and features, such as peak frequencies, bandwidth, spectral density, and cepstral coefficients (CC), of the recorded signals to pre-established profiles for different species and life stages [41] or to distinguish between insect and background noise [75].

Some insects can generate high-frequency ultrasonic signals. Males of rice moths, *Corcyra cephalonica*, make calls with sound pulses of 125,000 Hz, while Indian meal moths, *Plodia interpunctella*, produce pulses at 100,000 Hz using tymbal organs located on the ventral side of the abdomen [76].

Initial signal filtering can be used to isolate the frequency range of interest and expose the relevant signals while removing superfluous noise from analysis that can vary between sensors and environments [14,38]. A band-pass filter from 200 Hz to 10,000 Hz was used to visualize the waveforms of *Acanthoscelides obtectus* in common beans as well as *Prostephanus trancatus* and *Sitophilus zeamais* larvae and adults in 200 g of maize. The mean spectra profiles were constructed using 512-point time slices centered at the peak of the exposed impulses and a least-squares algorithm matched similar impulses. Impulses that failed to match within a preset least-squares threshold were classified as noise [41,77].

The propagation of the sound through the granular material is dependent on the substrate properties, including density, kernel size and hardness, relative humidity, and the amount of air gaps [38]. Two Brüel & Kjær 13 mm microphones measured the sound transmission of a speaker through 5 m^3^ of wheat, observing the material act as a low-pass filter, highly absorbing frequencies above 1000 Hz over a distance of 1 m. Average spacing between kernels decreased with depth in a bulk grain. Besides potentially making it difficult for insects to maneuver through, this would further create a gradient in the filtering effect. The reduced porosity decreases air gaps, altering how the acoustic waves travel through the material. This change in tortuosity, a metric that describes the porosity of the material and the complexity of the path the sound must travel through, would result in different distances for the sound waves to travel, altering scattering rates at grain interfaces, and energy dissipation as a result of the multiple reflections and refraction [78].

The numerous sensors have different sensitivities and a majority of them are not calibrated. Normalization methods adjust the amplitude of the signal to a common scale, enabling better comparison and analysis of signals across different recordings, conditions, and equipment as well as accommodating varying distances from the insect subject to the sensor. Although various publications normalize the insect amplitude for analysis, the methods are not always described in detail [48,72,79,80,81]. The study [82] used Z-score normalization, which standardizes every value in the collection of data so that the mean of all values is zero and the standard deviation is one. This method was applied for wood-boring insects.

In our publication [51], the system’s self-noise, an average of recordings taken when no insects were present, was used to normalize the signals. After filtering and enveloping the waveforms, they were then normalized by the root mean square (RMS) of the filtered and enveloped self-noise.

Advances in machine learning (ML) algorithms in recent years demonstrated new opportunities for insect detection, quantification, and classification. ML algorithms can analyze acoustic signals and identify patterns associated with insect activity as well as distinguish external noise, but they depend on large amounts of training data. The ML models can be trained on the raw audio signals or on extracted features, such as spectrograms, and the data can be provided in labeled or unlabeled forms [15,68]. These algorithms include various neural network classifiers such as convolution neural networks (CNN), probabilistic neural networks (PNN), perceptual learning prediction (PLP), decision trees and forests, hidden Markov models (HMM), support vector machines (SVM), and Bayesian classifiers [14,58,82,83]. Deep learning combines multiple layers of neural networks to learn the features and patterns of the data. Since machine learning algorithms are data-driven, the performance of the model might not generalize to new species or materials, if the training data is not diverse enough.

#### 1.3.5. Automated Acoustic Systems for Insect Detection in Stored Products

Automated acoustic insect detection systems would ideally be capable of performing real-time detection of insects with minimal human intervention. The performance sensors and associated algorithm are usually evaluated through their detection accuracy of correctly identifying infested and uninfested samples, the false positive rate of incorrectly identifying uninfested samples as infested, and the false negative rate of incorrectly identifying infested samples as clean.

One system featuring an acoustic microphone attached, but decoupled, to a grain container through a diaphragm and sealed air column achieved a 71% to 90% detection rate for *Sitophilus oryzae*, *Rhyzopertha dominica*, and *Sitotroga cerelella* in wheat. The signals were amplified by 60 dB, filtered in the range of 200 Hz to 8000 Hz, enveloped, and an amplitude threshold crossing algorithm determined the detection. The system and algorithm were able to estimate the infestation rates in the range of 1 to 20 kernels per 100 mL of sample [42,84].

Another system consisted of 16 piezoelectric sensors installed across four cables in a 208 L steel drum to monitor two to 20 adult *Tribolium castaneum* and *Rhyzopertha dominica* in 1.5 kg of wheat. Although the preliminary signal processing is not explicitly described, the authors observed a high correlation between the number of insects within 100 mL of wheat and the number of insect sounds detected over 60 s intervals. Using this correlation, the authors were able to fit a linear regression model and achieve a 90% detection rate for densities from 0.04 to 0.32 insects per 1 kg [85].

The Acoustic Location Fixing Insect Detector (ALFID) used an array of 16 piezoelectric sensors distributed vertically in a gravity-loaded, 76 cm by 5 cm by 4 cm, container capable of testing 1 kg of sample. A band-pass filter from 1000 Hz to 10,000 Hz was applied to the signals and an amplitude threshold detection was used for detection. The system allows estimation of insect location based on the sensor position with maximal signal amplitude. Larvae of the *S. oryzae* in 1 kg of wheat were detected with a 64% accuracy and a false positive rate of 8%. Clean wheat samples were correctly identified as uninfested with a 92% accuracy [50,86]. The system was patented in 1996 [87].

The Early Warning Detector incorporated three levels of piezoelectric sensors into a 1.4 m probe that can be inserted into grain bin units. The algorithm compared the insect signal spectra to a previously collected reference database of 990 samples. The system was able to detect adults and larva of *S. oryzae* and *R. dominica* at infestation levels ranging from one to ten insect larva per 10 kg of grain with a confidence factor of 95% [47].

A 14 cm long piezoelectric sensor mounted on a probe was used to collect signals of wheat and corn inhabited by *Sitophilus oryzae*, *Rhyzopertha dominica*, *Tribolium confusum*, *Oryzaephilus surinamensis*, *Cryptolestes ferrugineus*, and *Lasioderma serricorne* at different infestation levels. After amplifying the signals through an Acoustic Emission Amplifier (AED-2010L), the impulses were extracted using a Hilbert transformation and a threshold crossing algorithm determined a positive detection when the envelope exceeded three times the mean of 80% of the lowest amplitudes. The system achieved a 100% negative detection rate and a 72% to 100% positive detection rate for one to two insects per kilogram depending on the species [48]. In a following publication, the authors utilized the same system and compared different machine learning algorithms to determine infestation levels with a success rate above 70% [88].

The Portable Postharvest Detection System (PDS) utilized electret microphones embedded in a plexiglass base. After a band-pass filter was applied in the range of 20 Hz to 5000 Hz, spectral matching software was used, specifically in the frequencies in the range of 500 Hz to 2500 Hz, to compare matching sound spectra, sound rate, and other likelihood indicators to a database of known insect sounds and discriminate against background noise. The system detected *Sitophilus oryzae* adults in 2.6 kg of grain at a rate of 1.9 insects per kg and *Tribolium castaneum* adults at a rate of 3.8 insects per kg of flour [40].

The Beetle Sound Tube system consisted of perforated tubes placed vertically into grain bins with an insect trap at the bottom. A precision piezoelectric microphone was used for acoustic signal recording. The system was scalable and was demonstrated in ranges from a single tube in a 1 L container, three tubes in a 300 t silo, to nine tubes in a flat storage container. The system’s performance was detailed for *Oryzaephilus surinamensis* in barley. The underlying algorithm of the system collected 5 min of acoustic data every hour, divided it into blocks of 128 samples with a duration of 2.7 ms, and calculated the energy level of each block. After setting the minimum energy in a 5 min recording as the background energy level, any energy blocks that exceeded this level by 5 dB were used to determine the beginning and end of a pulse, resulting in a standardized pulse length of 1280 samples. A detection algorithm using $\frac{1}{3}$-octave band analysis enabled instantaneous detection by calculating the energy across 16 different bands in the range of 250 Hz to 6000 Hz and comparing it to the background noise level, which was determined as 5% quantile of the energy level for each band. A signal was counted as an insect event if its energy exceeded frequency-specific thresholds, in the range of 15 dB to 25 dB above background noise within bands at 800 Hz or higher, while signals exceeding the threshold below 800 Hz were discarded as noise [81].

Another system used a piezoelectric sensor inserted through a 5 cm hole into a 60 cm by 30 cm by 37 cm bin capable of holding 10 kg of material and insulated with 20 mm aluminum alloy and 40 mm polyurethane foam with a noise reduction coefficient (NRC) of 0.80. The sounds of ten individual *Callosobruchus chinensis*, *Zabrotes subfasciatus*, *Callosobruchus maculatus*, and *Centris analis* feeding as larvae and moving as adults in red and green beans were recorded in varying temperatures in the range of 10 °C to 38 °C. A Finite Impulse Response (FIR) high-pass filter was applied with a cutoff frequency of 2000 Hz. The number of pulses, their sizes, and durations were measured to determine that the optimal temperature was 31 °C and that pulse duration was the best characteristic to distinguish between species regardless of temperature and bean type [49].

A 100% detection rate was achieved for *Sitophilus oryzae* in rice using a system with a piezoelectric sensor described in [83]. After applying a high-pass filter at 2000 Hz, the linear frequency cepstral coefficients (LFCC) and discrete wavelet packet features (DWPF) were extracted from the signals. A Gaussian Mixture Model (GMM) classifier was trained on the features to determine the presence of insects.

Micro-electromechanical system (MEMS) microphones, the Adafruit I2S SPH0645, were used to measure the sounds of *Rhyzopertha dominica*, *Sitophilus oryzae*, and *Tribolium castaneum* in a 16 cm by 16 cm by 8 cm container filled with 500 g sample of rice. After applying a band-pass filter in the range of 93.75 Hz to 3000 Hz, the authors found identifiable differences in peak frequencies between the species as well as in the root mean square (RMS) energy. A convolutional neural network (CNN) model was trained on generated spectrogram images of the signal and achieved a 84.51% classification accuracy [58,89].

The regularity and duration of the monitoring also have an effect on the performance of the sensor and its algorithm. The *Rhyzopertha dominica*, *Sitotroga cerealella*, and *Sitophilus oryzae* produced sounds 61%, 71%, and 90% of the time in grain during sampling durations of 180 s [42]. A short analysis duration can increase rates of false negatives, especially if the insects are not active during the sampling period. Longer sampling periods, such as 10 min to 20 min allow for a more accurate detection as well as enable identification of background noise [38]. Deployment of multiple sensors simultaneously could increase detection performance and determine locations of insects and the level of infestation, and to offset the frequency and duration of monitoring [38].

Table 1 summarizes the performance of several automated acoustic detection systems for stored product insects including the tested subject-material, sensor type, subject-material concentrate, noise suppression methods, insect detection algorithm, and reference.

### 1.4. Knowledge Gaps and Research Objectives

Despite the development of numerous acoustic insect detection systems for stored products, their practical application remains limited. Lightweight sensors are highly susceptible to external noise and vibration, whereas systems incorporating heavy soundproofing are too cumbersome for field deployment. Development of a reliable, low-weight acoustic system for stored product insect detection requires investigations of the following research problems:

- Understanding of acoustic signatures of different insects in various materials.
- Detailed measurements of the acoustic properties of soundproofed enclosures for optimized noise attenuation.
- Analysis of the external noise influence on sensor signals.
- Development of an insect detection algorithm that includes application of an external microphone for ambient noise suppression.

The early prototype of the Acoustic Stored Product Insect Detection System (A-SPIDS) was developed to support these objectives and the first tests were conducted for the collection of acoustic insect signatures [51]. This system offers a significantly reduced weight and size compared to the previously reviewed soundproofed chambers, enabling its use for field deployment, particularly at U.S. ports of entry. The innovation of the presented work is the development and testing of the insect detection algorithms for A-SPIDS that are workable in noisy environments. These algorithms rely on measured insect acoustic signatures and their comparison with externally generated noise penetrating through the sound-insulated enclosure. The advanced iteration of the algorithm utilizes the external microphone incorporated in the A-SPIDS platform design to suppress the influence of external noise and improve the detection performance of the system.

## 2. Materials and Methods

### 2.1. Acoustic Stored Product Insect Detection System (A-SPIDS)

The Acoustic Stored Product Insect Detection System (A-SPIDS) was designed as a low-cost, non-destructive, and portable inspection system. The tabletop platform comprises three components. The main component is a box-in-a-box sampling container capable of handling approximately 1.5 L of dry storage product housed within a chamber outfitted with insulation and data acquisition electronics. The system is powered by a rechargeable battery and transmits the data wirelessly to a computer. The sampling container rests on a base that provides wireless charging and vibration isolation. A removable lid allows for easy access to the sampling container and can be securely closed to create a soundproof seal.

**Figure 1:**
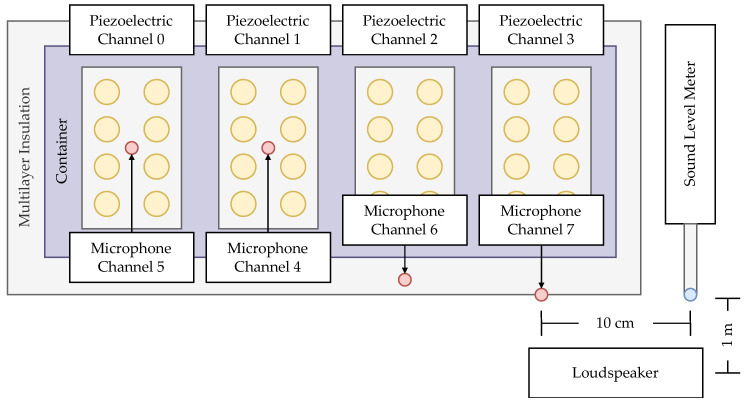
Diagram of the A-SPIDS platform showing the piezoelectric sensors, acoustic microphones, and the locations of the sound level meter and loudspeaker during external noise testing.

The bottom of the testing receptacle is lined with 32 piezoelectric discs (model AB2746B, PUI Audio, Hong Kong, China) arranged in four groups of eight corresponding to channels zero through three in the recordings. The 27 mm diameter and 0.51 mm thick brass discs have a resonant frequency of 4600±500 Hz, a capacitance of 16 nF ± 30% at 1000 Hz, a maximum resonant impedance of 250 Ω, and a maximum input voltage rating of 20 Vp-p [90]. Figure 1 depicts the placement of the piezoelectric sensors in the system.

**Figure 2:**
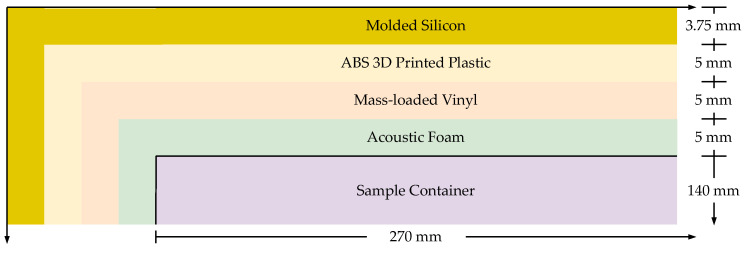
Diagram of the A-SPIDS container component insulation cross-section.

The sides of the container and the bottom below the sensor array are insulated with a layer of 5 mm of acoustic foam (Quiet Barrier Soundproof Material, Soundproof Cow, Chambersburg, PA, USA) [91] and 5 mm layer of mass-loaded vinyl (MLV) (Quiet Barrier Speciality Composite, Soundproof Cow, Chambersburg, PA, USA) [92]. Figure 2 depicts the cross-section of the container and the placement of the insulation layers.

The top perimeter of the container and the full bottom surface of the lid are lined with 3.75 mm of molded silicon [93] to create a tight seal when the lid is closed and to further reduce external noise and vibration. The body of the lid and the base are also filled with the same acoustic foam and mass-loaded vinyl layers. The external shell of the full system is made of 3D-printed acrylonitrile butadiene styrene (ABS) plastic. The A-SPIDS platform weighs 9 kg with a length, height, and width of 30 cm, 24 cm, and 19 cm, respectively.

Traditional acoustic microphones (model P OM-2735P-R, PUI Audio, Hong Kong, China) with a diameter of 6 mm, a height of 4.7 mm, and a sensitivity of −35±2 dB [94], are placed throughout the system to capture the external noise and vibration. The four microphones transmit through channels four to seven, respectively. Microphone channels four and five are collocated with the piezoelectric channels zero and one, respectively, while channel six is placed within the acoustic foam, and channel seven is mounted on the exterior of the system. The external microphone was used for ambient noise reduction, while the internal microphones were applied for investigations of sound suppression by the soundproofed enclosure and were not used in the insect detection algorithm. Figure 1 depicts the placement of the acoustic microphones in the system. Further details on the system design are described in a previous publication [51].

### 2.2. Tested Insect, Materials, and Noise Levels

Although stored product insects proliferate across a wide range of commodities, they are difficult to obtain for research purposes. The cowpea beetle, *Callosobruchus maculatus*, the confused flour beetle, *Tribolium confusum*, and the mealworm beetle, *Tenebrio molitor*, are commercially available and were chosen based on their similarities in size, qualities, and behavior to the species targeted during inspection. Insects were obtained from local pet supply stores and reared in laboratory conditions ranging from 22 °C to 27 °C with a relative humidity of 40% to 60%. The *C. maculatus*, *T. confusum*, and *T. molitor* were reared in cowpeas, wheat flour, and oats, respectively. Adult insects at various ages were used during testing except for the *T. molitor*, where larvae were used as well.

A variety of stored product materials were tested with and without insect specimen, including oatmeal, *Avena sativa*, rice, *Oryza sativa*, wheat in the form of whole groat kernels and processed flour, *Triticum aestivum*, and corn in the form of Corn Flakes, *Zea mays*. The material samples were kept in sealed storage containers to prevent contamination and infestation. During testing, 1 L of clean material was transferred into a plastic bag and placed into the sensor container. After several minutes of silence were recorded to ensure the grains were settled, a single insect was introduced into the material.

Testing was conducted in varying levels of ambient and simulated noise. The ambient noise was a result of the surrounding laboratory environment, including equipment and HVAC units, as well as vehicular and aircraft traffic. Artificial noise, such as pre-recorded sweeps, white noise, single-person speech, crowds, and factory noise, was played through a loudspeaker at varying sound levels in the range of 60 dBA to 100 dBA at 10 dB increments and controlled using the calibrated Sound Level Meter (model Extech 407730, Teledyne FLIR, Wilsonville, OR, USA) [95] collaterally placed approximately 10 cm from the external sensor. The loudspeaker was placed 1 m from the system and SPL meter. Figure 1 depicts the A-SPIDS platform and the placement of the external microphone and SPL meter during testing.

### 2.3. Acoustic Stored Product Insect Dataset (ASPID)

The Acoustic Stored Product Insect Dataset (ASPID) is a repository of audio recordings collected by the Acoustic Stored Product Insect Detection System and made publicly available for download from Kaggle [96]. At the time of publication, the dataset contains almost 37 h of recordings of different insect–material pairings and also features control recordings of clean materials without insects. The recordings are conducted in silence as well as with ambient and simulated noise. Table 2 describes the duration of recordings available in the dataset for each insect–material pairing and no insect control. Recordings with external noise are indicated in parentheses.

In the paper [51], a subset of the Acoustic Stored Product Insect Dataset was extracted for investigation of insect signatures. The subset features 1 min recordings of different insect–material pairings, in silence, when an insect was present and observed moving with at least five impulses in acoustic visualization figures. Table 3 summarizes the number of records for each insect–material pairing in the subset. The subset contains a total of 1329 recordings, including five control recordings without insects but with simulated, insect-like, impulse noise ranging in the range of 60 dBA to 100 dBA. Details on extracting the subset from the database are provided in the associated repository [97]. This subset is used for analysis in this paper as well.

### 2.4. Characterization of Acoustic Signatures Generated by Insects in Stored Products

The paper [51] considered two parameters for insect acoustic signature characterization, the Normalized Signal Pulse Amplitude (NSPA) and the Normalized Spectral Energy Level (NSEL). The normalization was conducted by the system self-noise when no insects were present. These parameters characterized the signal-to-noise ratio (SNR) for pulse-based detection and average energy-based detection, respectively. In the processed data, the NSPA metric had a higher magnitude and was determined to be a more effective method for a threshold-based detection algorithm.

The NSPA estimates the average amplitude of acoustic pulses generated by insect activity. It is calculated for each channel by first applying a Butterworth band-pass filter to the respective signal. A Butterworth band-pass filter is able to provide a flat frequency response in the pass-band while attenuating frequencies outside of the desired range [98]. Equation (1) describes the magnitude response function of this filter:

(1) $$M\left(f\right)=\frac{1}{\sqrt{1+{\left(\frac{f_{c}^{2}-f^{2}}{f\;\cdot \;f_{\Delta }}\right)}^{2n}}}\;f_{c}=\sqrt{f_{1}\;\cdot \;f_{2}}\;f_{\Delta }=f_{2}-f_{1}$$

where $f_{c}$ is the center frequency, $f_{1}$ and $f_{2}$ are the lower and higher frequency boundaries, respectively, and *n* is the order of the filter [99]. Next, the envelope of the filtered signal is extracted using the Hilbert transform. The Hilbert transform, *H*, of the signal S(t) is defined in Equation (2):

(2) $$\hat{S}\left(t\right)=H\left\{S\left(t\right)\right\}=\frac{1}{\pi }\int_{-\infty }^{\infty }\frac{S(\tau )}{t-\tau }d\tau$$

where *t* is the time and τ is the integration variable. Equation (3) shows the analytical signal, Z(t), obtained by combining the original signal with the Hilbert transform:

(3) $$Z\left(t\right)=S\left(t\right)+i\hat{S}\left(t\right)$$

The envelope of the signal is then calculated by taking the magnitude of the analytical signal, Z(t), as shown in Equation (4):

(4) $$S_{E}=\left|z\left(t\right)\right|=\sqrt{S^{2}+{\hat{S}}^{2}}$$

The filtered signal envelope is then normalized by the sensor self-noise recording obtained when no insect is present. The normalization process involves applying the same band-pass filter and envelope extraction to the self-noise recording. The normalized signal, $S_{c}$, for channel *c* is calculated by dividing the filtered envelope of the insect recording, $S_{E,c}$, by the root mean square (RMS) of the filtered envelope of the self-noise recording, $N_{E,c}$, as shown in Equation (5):

(5) $$V=\frac{S_{E,\mathrm{BPF}(f_{1},f_{2})}}{\mathrm{RMS}(N_{E,\mathrm{BPF}(f_{1},f_{2})})}$$

where the normalized signal amplitude *V* is the envelope of the band-pass filtered signal $S_{E,\mathrm{BPF}}$ divided by the root mean square of the enveloped band-pass filtered reference self-noise $N_{E,\mathrm{BPF}}$ in the frequency band from $f_{1}$ to $f_{2}$. An analysis region, where the impulses generated by the insect activity have the highest amplitudes, is determined as 50% to 90% of the maximum envelope amplitude. Finally, the NSPA is expressed in decibels. Equation (6) describes the NSPA calculation:

(6) $$\mathrm{NSPA}=20\log_{10}\left(\mathrm{RMS}\left(V\right)\right)\;\mathrm{where}\;0.5V_{\max}\leq V\leq 0.9V_{\max}$$

where *V* is the filtered envelope of the normalized signal, RMS is the root mean square function, and $V_{\max}$ is the maximum envelope amplitude of the analyzed record.

**Figure 3:**
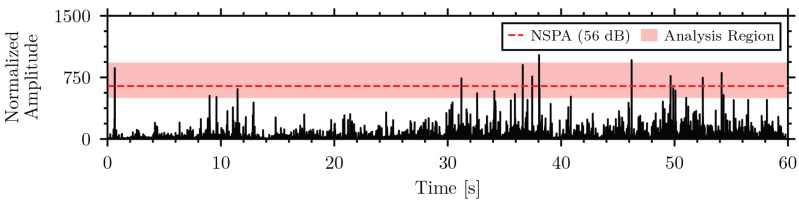
Normalized filtered envelope, *V*, of *T. molitor* in rice with NSPA-calculated region highlighted and value indicated.

Spectral analysis of the insect recordings determined that the majority of the insect signal energy was concentrated in the 500 Hz to 6000 Hz frequency range [51]. Therefore, the band-pass filter was set to these cutoff frequencies for the NSPA calculation. A recording duration of 1 min was selected to balance the need for a quick detection while allowing sufficient time for insect activity to occur. Figure 3 shows a normalized filtered envelope of the piezoelectric sensor recording of *T. molitor* in rice with the analysis region highlighted and the NSPA value indicated.

## 3. Results

### 3.1. Normalized Signal Pulse Amplitude (NSPA) as a Criteria for Insect Detection

The NSPA as a statistical value was calculated for each insect–material pairing in the subset described in Table 3, resulting in a range from 27 dB to 75 dB [51]. This parameter can be used as a detection criterion when it exceeds a definite threshold, *T*, depending on the external noise considered acceptable for the application. If NSPA≥T, then an insect is detected; otherwise, no insect is detected. Setting the detection threshold, *T*, to the minimum observed value of 27 dB results in nearly 100% detection of the tested insects. It should be emphasized, however, that this performance was achieved under quiet laboratory conditions. The subsequent sections examine the influence of ambient noise and describe adaptations to the detection algorithm for operation in noisy environments.

### 3.2. Generation of Insect-like Signals by External Noise

The developed acoustic insect detection system provided sufficient physical soundproofing that enabled measurement of insect signatures in laboratory conditions with high SNR by the integrated piezoelectric sensors without interference from external noise.

**Figure 4:**
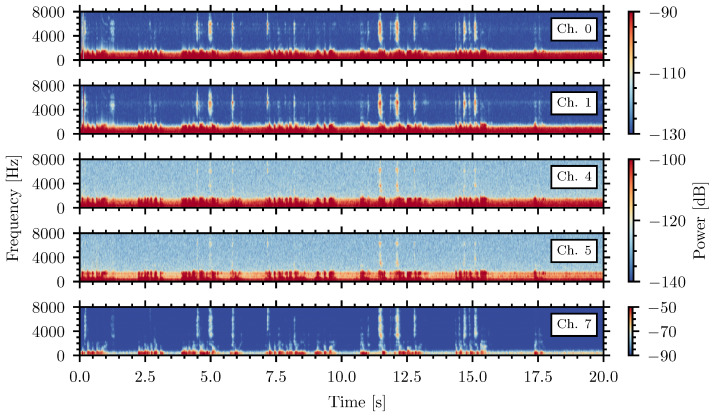
Spectrogram from internal piezoelectric sensors, channel zero and one; internal acoustic microphones, channels four and five; and external microphone, channel seven, during external amplified speech at 90 dBA that exceeds the level of normal speech. Insect-like pulses are visible in all channels.

Strong external noise with impulse characteristics, however, can still penetrate inside the enclosure and generate signals similar to those produced by insects, mask their presence, and generate false alarms. This is highlighted in Figure 4, which shows the spectrograms of the recordings from the internal piezoelectric sensors, channels zero and one; the collocated microphones, channels four and five; and the external microphone, channel seven, during external amplified male speech at 90 dBA. The amplitude color scales of the piezoelectric and microphone spectrograms are adjusted and differentiated to highlight the impulses generated by the external sound.

Various kinds of noise can produce different peak levels for the same averaged sound level. The insect-like signals generated by the noise peaks, as seen in Figure 4, would result in more false alarms than monotonic noise. The crest factor parameter characterizes the impulsive nature of the signal through its peak-to-RMS ratio [100]. The expected noises at U.S. Ports of Entry, such as machinery and vehicles, generate continuous signals with low crest factors in the range of 1 dB to 3 dB [101,102], and impulsive noises, such as hammering and pneumatic tools, with higher crest factors in the range of 5 dB to 10 dB [101]. The majority of the industrial noise also has lower frequency content [103] and the significant part of its energy would be suppressed through filtering in the developed algorithm. Analysis of the recorded signals, after normalized filtered envelope processing, of factory noise [104,105] and male speech showed a crest factor of 8 dB and 13 dB, respectively. This demonstrated that the high crest factor speech recordings would be more disruptive to the insect detection algorithm than the industrial noise at the same sound level. If the developed algorithm allows insect detection at noise levels of 100 dBA for the speech noise recording, it would be expected to perform well in the presence of industrial noise even at higher sound levels.

**Figure 5:**
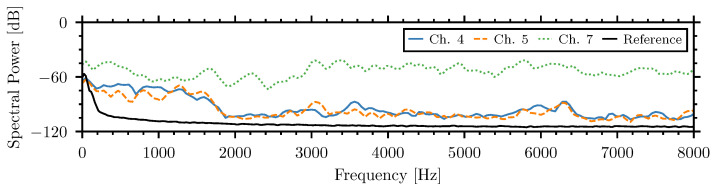
Spectra of strong pulses recorded by internal microphone, channel four and five, and external microphone, channel seven, during 90 dBA external noise.

The spectrograms show the variation in the external signal as it penetrates to the piezoceramic sensors and internal microphones. The external microphone, channel seven, shows the signal in air and highly exceeds the magnitude levels compared to the internal sensors. The impulses are similar to the insect signals presented in Figure 5 of the previous publication [51]. The NSPA values of the piezoelectric sensors calculated from the noise recordings in the range of 39 dB to 69 dB at noise levels in the range of 60 dBA to 100 dBA, respectively, which are comparable to or exceed those of the insect signals. This demonstrates that the detection algorithm based on NSPA could report a false positive, an insect detection when no insect is present.

Measurements of sound attenuation provided by the soundproofed enclosure require the application of strong acoustic signals that can penetrate through the insulation. For this purpose, the strongest parts of the external noise were analyzed. In Figure 4, pulses observed by the internal microphones, channels four and five, are approximately 50 dB lower than those recorded by external microphone seven. This large drop in the acoustic signal magnitude within the soundproofed chamber demonstrates the effectiveness of the sound reduction the system. Figure 5 presents the spectra of the impulse with the maximal amplitude calculated in the short time window of 100 ms for acoustic microphones four, five, and seven in the presence of the external speech noise with a level of 90 dBA. The reference signal of the averaged self-noise from the internal microphones, channels four and five, is also shown.

The difference in the spectral power between the microphones demonstrates the effectiveness of the insulation in muffling external noise, especially at frequencies above 2000 Hz, where it was able to provide an average of 45 dB and a maximum of 59 dB noise reduction. This level of attenuation is comparable to the results achieved with the 620 kg chamber used for the InsectSound100 dataset but in a more compact and portable form factor [44,64].

**Figure 6:**
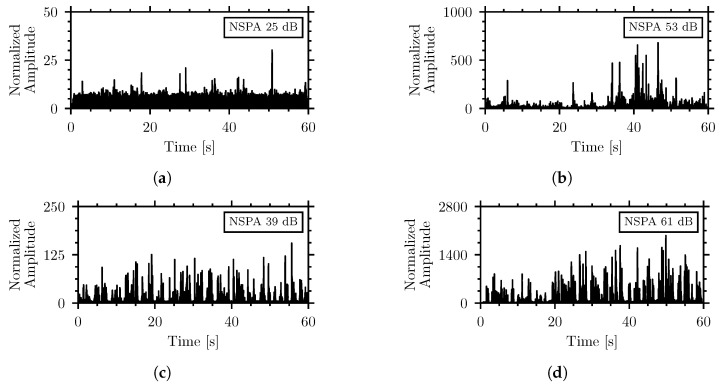
Filtered, enveloped, and normalized waveforms (500 Hz to 6000 Hz) of (**a**) weak strength insect signal of *T. confusum* in flour, (**b**) medium insect signal of *C. maculatus* in wheat groats, (**c**) signal of 60 dBA external speech noise, (**d**) signal of 90 dBA external speech noise. The graphics are accompanied by their NSPA value calculated in the same frequency range.

The high sensitivity of the piezoelectric sensors makes them vulnerable to external noise even through the insulation. The filtered envelope of the normalized signal can visualize the waveforms of the acoustic recordings from the system. Figure 6 shows the waveforms, filtered, enveloped, and normalized in the 500 Hz to 6000 Hz frequency range, for two examples of weak and medium insect signals, the *T. confusum* in flour and the *C. maculatus* in wheat groats, respectively, as well as the speech noise at 60 dBA and 90 dBA. The graphics are accompanied by the NSPA values calculated over the same frequency range.

The waveforms show that the generated pulses from the external noise reach similar amplitudes to those from insect activity. Even at 60 dBA, in Figure 6c, the impulse amplitudes and NSPA highly exceed those in Figure 6a of the *T. confusum* in flour. The medium-strength insect signal, in Figure 6b, has a lower amplitude and NSPA compared to the noise at 90 dBA in Figure 6d. This further demonstrates that the NSPA-based detection algorithm in the considered form is not applicable here since the external noise can generate insect-like signals that exceed those from actual insect activity.

**Figure 7:**
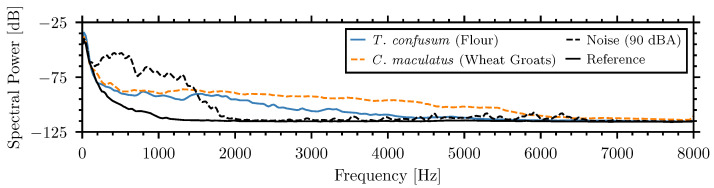
Spectral power of *T. confusum* in flour, *C. maculatus* in wheat groats, and external speech noise at 90 dB recorded by channel zero.

Figure 7 compares the spectra, averaged over 1 min, recorded by the internal piezoelectric sensor of the weak and medium insect signals with the speech noise at 90 dBA. While external noise exhibits dominant spectral power below 1600 Hz, insect signals demonstrate higher energy content above this frequency.

These spectra show that noise highly exceeds insect signals in the frequencies below 1500 Hz but insect signals have higher energy content at higher frequencies. Adjusting the frequency band of the system could decrease the influence of external noise on the piezoelectric sensors. This adjustment was made based on the frequency dependence of the NSPA for insect and noise signals.

The lower and upper frequency limits of the NSPA algorithm were parameterized in order to optimize the differentiation between insect and noise signals. Twenty samples of the insect signals representative of the median strength for each respective insect–material pairing were selected for analysis. The frequency bands were systematically varied and the respective NSPA values were calculated and compared to the NSPA values of the external noise at 90 dBA to maximize the difference.

**Figure 8:**
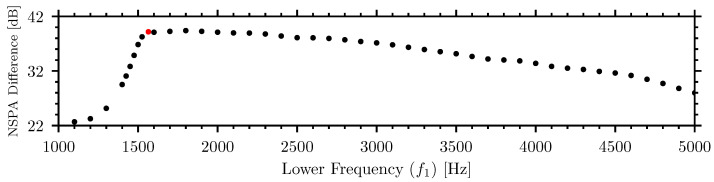
Dependence of NSPA difference between insect and external noise signals on the lower frequency, $f_{1}$, of the band-pass filter. The optimal low frequency, 1565 Hz, is highlighted in red.

The NSPA difference significantly increased when the lower frequency of the band-pass filter was above 1500 Hz. The upper frequency limit plays a less critical role. Focusing on the lower frequency range, Figure 8 shows the NSPA difference between the insect and noise signals as the lower frequency bound is varied in the range of 1000 Hz to 5000 Hz while the upper frequency bound is held constant at 6000 Hz.

The optimal low frequency, when the difference between the insect and noise NSPA is maximized, converged to 1565 Hz before it started to decrease as a result of losing insect signal energy at higher frequencies. The frequency range for filtering and normalization was adjusted to 1565 Hz to 6000 Hz.

**Figure 9:**
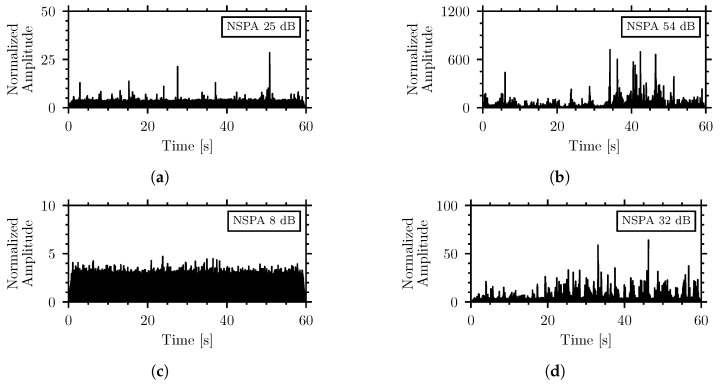
Normalized envelope of signal filtered in band 1565 Hz to 6000 Hz recorded by the internal piezoelectric of (**a**) weak strength *T. confusum* in flour, (**b**) medium strength *C. maculatus* in wheat groats, (**c**) 60 dBA external speech noise, and (**d**) 90 dBA external speech noise.

**Figure 10:**
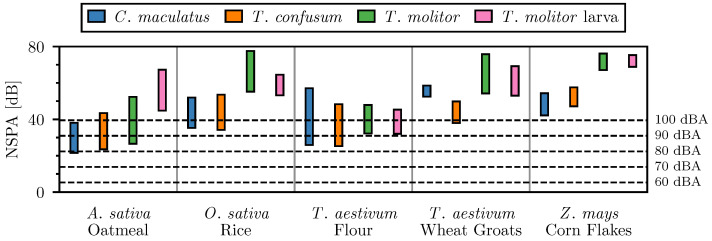
Distribution of NSPA values for different insect–material pairings and external noise.

Figure 9 shows the filtered and normalized waveforms for the same recordings as Figure 6 but using the updated frequency range:

Comparison of Figure 6 and Figure 9 shows that adjustment of the low cutoff frequency significantly decreases the NSPA produced by external noise. The NSPA values of the different insect–material pairings and external noise recordings were calculated using the frequency range of 1565 Hz to 6000 Hz. Figure 10 shows the distribution of the NSPA values using the adjusted frequency range. Accompanying the box plot are horizontal lines representing the NSPA values measured by the piezoelectric sensors at different external noise levels in the range of 60 dBA to 100 dBA.

The updated frequency range significantly improved the NSPA difference between the insect signals and the external noise. At a noise level of 80 dBA, nearly all measured NSPA values exceed those generated by external noise. The resulting NSPA range of the insect–material pairings now extends in the range of 22 dB to 78 dB. If the threshold, *T*, is set to the maximum NSPA value of the external noise at 80 dBA, 22 dB, 99.4% of the insect–material pairings would be correctly identified as containing an insect. At this threshold, any external noise above 80 dBA could generate false positive insect detections. Increasing the threshold to the maximum NSPA value of external noise at 90 dBA, 31 dB, would result in 90.2% correct insect detections.

### 3.3. Leveraging External Acoustic Microphone for Noise Control

The previous sections demonstrated that even single small insects can be detected by the developed system using the NSPA threshold algorithm under speech noise sound levels up to 80 dBA. An additional level of noise protection can be achieved by using noise signal recording by an external microphone. The suggested external microphone noise control can allow insect detection in noisy conditions and can be used for insect detection in large volumes of products where the insect signals could be small due to longer propagation paths.

A coefficient, *K*, can be defined to describe the penetration of the external sound to the piezoelectric sensor, representing the ratio between the insect-like signals recorded by the internal piezoelectric sensors and the signal from the external microphone. The average value of the penetration coefficient, *K*, was found to be approximately 0.03, or −31 dB, across the four channels and five external noise levels in the range of 60 dBA to 90 dBA. The microphone signal can be scaled by this coefficient to estimate the signal on the internal piezoelectric sensors generated by the external noise, $V_{\mathrm{noise}}=KV_{\mathrm{mic}}$. When the measured NSPA(V) is greater than both $\mathrm{NSPA}(KV_{\mathrm{mic}})$ and the threshold, *T*, external noise does not influence insect detection. But if the estimated noise signal exceeds the threshold, $\mathrm{NSPA}(KV_{\mathrm{mic}})>T$, the ambient noise can generate a false positive detection even when no insect is present and a noise suppression algorithm can be applied.

The noise suppression algorithm identifies short time windows where external noise generates signals with amplitudes that are close to or exceed the threshold amplitude and can affect the NSPA measurement. Equation (7) describes the amplitude threshold limit:

(7) $$A_{T}=10^{\frac{T}{20}}$$

Peaks in the microphone signal with an amplitude, *V*, that exceeds $A_{T}/2K$ are identified. A time window of 10 ms centered around each peak is used to search for corresponding peaks in the piezoelectric sensor waveforms. If a peak is found within this time window, it is zeroed out. The NSPA calculation is then applied after the noise removal process.

**Figure 11:**
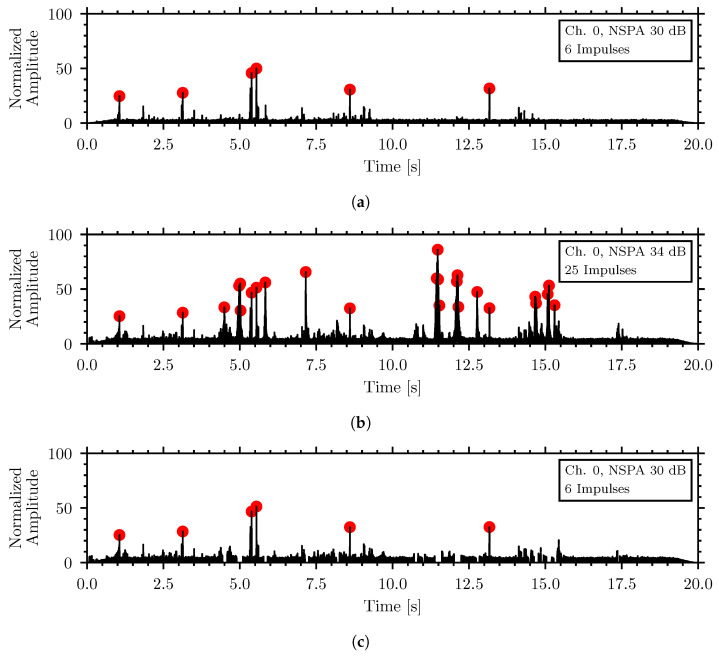
Simulation of external noise algorithm with 90 dBA speech noise and *T. confusum* in flour (**a**) insect signal, (**b**) combined insect signal and external noise, (**c**) combined signal after the noise removal algorithm.

Figure 11 demonstrates the application of the algorithm with the 90 dBA external speech noise shown in the previous figures combined with a recording of a relatively weaker insect signal of *T. confusum* in rice. Figure 11a shows the insect waveform, before adding noise. Figure 11b shows the combined waveform of the external noise and insect signal, where the external noise produces numerous additional insect-like signals that prevent insect detection without an application of a noise removal algorithm. Figure 11c shows the cleaned waveform after applying the noise removal algorithm. The NSPA of the cleaned waveform is 30 dB with the six original insect peaks preserved, while the 19 additional noise-generated peaks have been removed.

**Figure 12:**
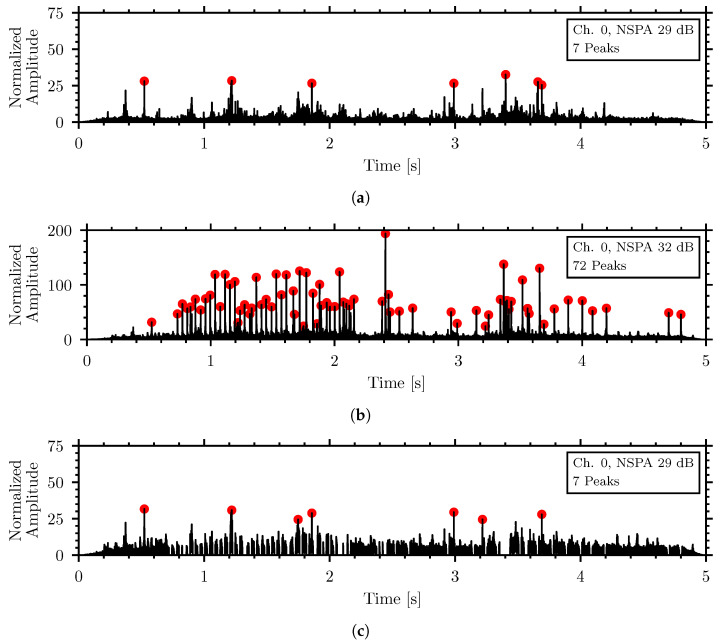
Simulation of external noise algorithm with chainsaw noise from the EC-50 Dataset and *T. molitor* in oatmeal (**a**) insect signal before adding noise, (**b**) combined waveform of external noise and insect signal, (**c**) cleaned combined waveform after applying the noise removal algorithm.

Figure 12 shows another example of the noise removal algorithm, this time with a 5 s recording of a chainsaw, representing a high sound level scenario that would be encountered as machinery noise at inspection facilities, retrieved from the EC-50 Environmental Sound Classification Dataset [106]. The recording was adjusted by a ratio of its RMS compared to the RMS of the 100 dBA speech noise recording to represent acquisition by the external microphone, channel seven. A copy of the recording was suppressed by the penetration coefficient, *K*, to simulate the piezoelectric sensor, channel zero. Figure 12a shows the insect waveform, before adding noise, of a *T. molitor* in oatmeal with an NSPA of 29 dB and seven identified impulse peaks above the threshold. Figure 12b shows the combined waveform of the external noise and insect signal, with a higher NSPA and 72 impulse peaks. Figure 12c shows the cleaned waveform after applying the noise removal algorithm. The NSPA of the cleaned waveform is 29 dB with the seven original insect peaks preserved while the 65 additional noise generated peaks have been removed.

### 3.4. Proposed Inspection Procedure and Finalized Detection Algorithm

Deployment of the A-SPIDS platform can follow the proposed subsequent procedure to ensure reliable insect detection even in high external noise environments.

It is expected that the frequency band for filtering and normalization, $f_{1}$ and $f_{2}$, and the insect detection thresholds, *T*, are established based on preliminary tests with insects expected to be found in the inspected materials. The data presented in this publication can be used as a reference for setting these parameters.

At the start of inspection operations, the system is calibrated by recording several minutes of ambient noise without insects present to establish the normalization baseline and the penetration coefficient, *K*, specific to the environment.

**Figure 13:**
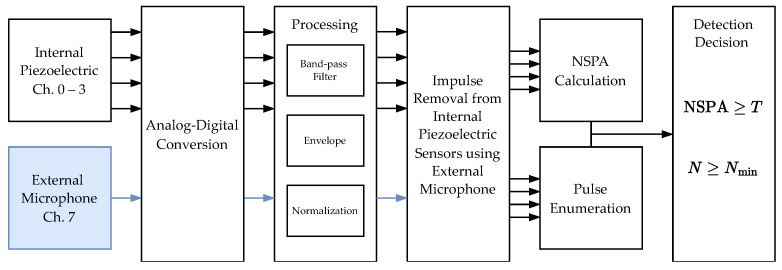
Flowchart of the signal processing, including the external noise removal algorithm and insect detection procedure. The microphone signal processing is marked in blue.

The operator places the sampled material into the container and after securing the lid, initiates the recording process. After a minute, during which the material settles, the recording session begins and continues for a predetermined duration, typically between one and five minutes, depending on the inspection requirements. The signals from the internal piezoelectric sensors, channels zero to three, and the external microphone, channel seven, are processed in real time during which they are filtered, enveloped, and normalized from $f_{1}$ to $f_{2}$. If impulses in the external microphone exceed the threshold, $\frac{A_{T}}{2K}$, the noise removal algorithm is applied to the internal piezoelectric sensor recordings. At one minute segments, the NSPA is calculated. Figure 13 diagrams the signal processing up to the insect detection decision:

The detection decision for each piezoelectric channel is made based on the NSPA value and the impulse count, *N*, after noise removal. If both criteria, NSPA≥T and $N\geq N_{\min}$, are satisfied, an insect is detected on that channel. If either criterion is not met, no insect is detected on that channel. The overall detection decision is made if any of the piezoelectric channels indicate the presence of an insect.

### 3.5. Perspective for Field Application

This work was supported by the United States Department of Homeland Security (DHS) Science and Technology (S&T) Directorate through the Cross-Border Threat Screening and Supply Chain Defense (CBTS) program and the developed prototype was considered as a step for the prospective prototype of a low-cost and non-destructive alternative to expedite the inspection of stored products at U.S. Ports of Entry.

Earlier, our group at Stevens Institute of Technology developed a prototype for detecting wood-boring insects in wooden shipping pallets, which was transferred to the United States Customs and Border Protection (CBP) for field testing. This prototype was based on the wood-boring insect detection algorithm presented in [107], but its description was not published.

The presented A-SPIDS prototype was tested in laboratory conditions, and investigating its performance within food-storage facilities and U.S. Ports of Entry is required. Field deployment at operational inspection facilities could provide critical data to refine the system design and algorithms based on real-world performance. Such deployments would yield insights into the variability of ambient noise profiles, substrate types and conditions, and insect behaviors encountered during routine inspection, as well as user feedback on the practical aspects of the system operation.

The perspective system could provide testing of larger volumes of material and include sensors to control external vibration. The high sensitivity and SNR of the system demonstrated its potential to handle higher volumes of material.

Additional features could be integrated into the algorithm to further enhance the detection accuracy and noise resilience. Implementation of machine learning approaches trained on the SPID dataset could provide more sophisticated detection and classification capabilities as well as adaptability to diverse noise conditions.

## 4. Conclusions

This study demonstrated the effectiveness of the Acoustic Stored Product Insect Detection System (A-SPIDS) for detecting a single insect in 1 L of stored product under challenging ambient noise conditions. The research addressed the critical limitation of acoustic detection methods, their vulnerability to external noise, by developing and validating a comprehensive algorithmic approach that enables reliable detection even in noisy operational environments.

The physical design of the A-SPIDS platform, featuring a box-in-a-box architecture with silicon and acoustic foam insulation, successfully attenuated external noise by a maximum of almost 59 dB and an average of 45 dB above 2000 Hz, achieving performance comparable to significantly larger and heavier systems while maintaining portability and practical deployment capabilities. The analysis also revealed that high-sensitivity piezoelectric sensors remained vulnerable to strong impulsive external noise that could penetrate the insulation and generate insect-like signals.

Spectral examination of insect signals against external noise revealed a critical distinction: while external noise exhibited dominant spectral power below 1600 Hz, insect-generated vibrations demonstrated higher energy content above this frequency. By optimizing the frequency band for the Normalized Signal Pulse Amplitude (NSPA) detection metric, 99.4% of the insect–material pairings in the dataset subset exceeded the maximum NSPA value generated by external noise at 80 dBA.

The additional layer of noise protection was provided by integrating the external microphone, which proved essential for noise control. The developed algorithm successfully identified and removed noise-generated impulses from the internal piezoelectric sensor recordings by exploiting the penetration coefficient relationship between external and internal sensors. The improved system achieved 100% insect detection with zero false positives across all tested conditions, including external noise levels up to 100 dBA.

## Figures and Tables

**Table 1:** Summary of automated acoustic detection systems.

Tested Subject–Material	Sensor Type	Volume of Tested Material and Noise Suppression	Insect Detection Algorithm	Reference
*S. oryzae*, *R. dominica*, and *S. cerealella*—wheat	Acoustic microphone	1–20 kernels per 100 mL placed in grain holder separated from sensor by diaphragm and sealed air column	Amplitude thresholding	[42,84]
*R. dominica*—wheat	Piezoelectric probe	2, 5, 10, 20 adults in 1.5 kg	Listening to amplified sound and pulse counting	[85]
*S. oryzae*—wheat	Piezoelectric disc array	1–3 larvae in 1 kg in a receptacle	Amplitude thresholding	[50,86]
*S. oryzae*, *R. dominica*—wheat	Piezoelectric probe	1–10 larvae and adults in 1 kg in sound insulated chamber	SNR and frequency peak position matching	[47]
*S. oryzae*—rice	Piezoelectric probe	Not disclosed	GMM classifier on FCC and DWPF features	[83]
*S. oryzae*, *R. dominica*, *T. confusum*, *O. surinamensis*, *C. ferrugineus*, and *L. serricorne*—wheat and corn	Piezoelectric probe	1–2 adults per 1, 2, 10 kg	Hilbert transform, envelope thresholding, machine learning	[48,88]
*T. inclusum*—rice	Piezoelectric array	1 adult in 1 kg within a box-in-box design with acoustic foam insulation	band-pass filtering and envelope thresholding	[80]
*S. oryzae* and *T. castaneum* in wheat and flour	Electret microphone	1–50 adults in 2.6 kg	Spectral matching	[40]
*O. surinamensis*—barley	Piezoelectric probe	70 t, 300 t, and 1 m^3^ containers, stored product material provides sound insulation	Energy thresholding in $\frac{1}{3}$-octave frequency bands	[81]
*R. dominica*, *S. oryzae*, and *T. castaneum*—rice	MEMS microphone	20 adults in 500 g placed in a double chamber with foam insulation and spring suspension	CNN on spectrogram images	[58,89]
*C. chinensis*, *Z. subfasiatus*, *C. maculatus*, and *C. analis*—red and green beans	Piezoelectric probe	10 adults in 10 kg placed in a bin insulated with aluminum and polyurethane foam	FIR high-pass filter and amplitude threshold	[49]

**Table 2:** Duration of recordings for each insect–material pairing, in minutes, available in the Acoustic Stored Product Insect Dataset. The duration of recordings with external noise is accommodated with parentheses.

	*A. sativa*	*O. sativa*	*T. aestivum*		*Z. mays*	Total
	Oatmeal	White Rice	Wheat Groats	Flour	Corn Flakes	
*C. maculatus*	12	258 (12)	13	20 (1)	17	320 (12)
*T. confusum*	39	274 (16)	189	55 (1)	16	573 (34)
*T. molitor*	68	15	9	8	13	113 (4)
*T. molitor* larva	46 (4)	268 (132)	51 (2)	128	43 (5)	536 (143)
No insect	4	219 (64)	142 (2)	24	23	412 (66)
Total	169 (4)	1034 (224)	403 (4)	235 (2)	112 (5)	1953 (239)

**Table 3:** Number of 1 min records for each insect–material pairing used for analysis.

	*A. sativa*	*O. sativa*	*T. aestivum*		*Z. mays*	Total
	Oatmeal	Rice	Wheat Groats	Flour	Corn Flakes	
*C. maculatus*	8	201	11	16	16	252
*T. confusum*	33	235	177	42	15	502
*T. molitor*	54	11	8	8	11	92
*T. molitor* larva	40	239	41	123	35	478
No Insect		5				5
Total	135	691	237	189	77	1329

## Data Availability

The data featured in this paper is publicly available on Kaggle as the A-SPIDS Stored Product Insect Dataset [96] and can be accessed using the Stored Product Insect Database available on GitHub at version 0.1 [97]. The code used for analysis and visualization is available on GitHub.

## Abbreviations

The following abbreviations are used in this manuscript:

A-SPIDS	Acoustic Stored Product Insect Detection System
ALFID	Acoustic Location Fingerprinting Insect Detector
ASPID	Acoustic Stored Product Insect Dataset
CBTS	Cross-Border Threat Screening and Supply Chain Defense
CNN	Convolutional Neural Network
dB	Decibel
dBA	A-Weighted Decibel
DHS	Department of Homeland Security
DWPF	Discrete Wavelet Packet Features
EAN	Emergency Alert Notification
EGPIC	Electronic Grain Probe Insect Counter
E-nose	Electronic Nose
EWD	Early Warning Detector
FIR	Finite Impulse Response
GC-MS	Gas Chromatography-Mass Spectrometry
GMM	Gaussian Mixture Model
HMM	Hidden Markov Model
IA	Impact Acoustics
ISO	International Standards Organization
LDV	Laser Doppler Vibrometer
LFCC	Linear Frequency Cepstral Coefficients
MEMS	Micro-Electromechanical Systems
ML	Machine Learning
NIR	Near-Infrared
NRC	Noise Reduction Coefficient
NSEL	Normalized Signal Energy Level
NSPA	Normalized Signal Pulse Amplitude
PD	Probability of Detection
PDS	Portable Postharvest Detection System
PFN	Probability of False Negative
PFP	Probability of False Positive
PLP	Perceptual Learning Prediction
PNN	Probabilistic Neural Network
RMS	Root Mean Square
S&T	Science and Technology
SNR	Signal-to-Noise Ratio
SPIDB	Stored Product Insect Database
SVM	Support Vector Machine
TNAU	Tamil Nadu Agricultural University
TOF	Time of Flight
VOC	Volatile Organic Compound
